# Integrated *in silico* and experimental evaluation of enhanced antioxidant effects of *Mentha* species using mixture design methodology

**DOI:** 10.3389/fchem.2026.1848118

**Published:** 2026-07-15

**Authors:** Meryem Tourabi, Mohamed El fadili, Naoufal El Hachlafi, Abdelouahid Samadi, Samir Chtita, Badiaa Lyoussi, Elhoussine Derwich

**Affiliations:** 1 Laboratory of Biotechnology, Conservation and Valorization of Bioresources, Faculty of Sciences, Sidi Mohamed Ben Abdellah University, Fez, Morocco; 2 LIMAS Laboratory, Faculty of Sciences Dhar El Mahraz, Sidi Mohamed Ben Abdellah University, Fez, Morocco; 3 Faculty of Medicine and Pharmacy, Ibn Zohr University, Guelmim, Morocco; 4 Department of Chemistry, College of Science, United Arab Emirates University, Al Ain, United Arab Emirates; 5 Laboratory of Analytical and Molecular Chemistry, Faculty of Sciences Ben M’Sik, Hassan II University of Casablanca, Casablanca, Morocco; 6 Unity of GC/MS and GC-FID, City of Innovation, Sidi Mohamed Ben Abdellah University, Fez, Morocco

**Keywords:** antioxidant activity, essential oils, food and pharmaceutical industry, *Mentha aquatica*, *Mentha longifolia*, *Mentha suaveolens*, mixture design, pharmacokinetics

## Abstract

The search for strongly effective and safe antioxidants is still one of the most important objectives targeted by the food and pharmaceutical industry. The growing concerns about the risks posed by synthetic additives have led to research into natural sources of antioxidants, especially essential oils (EOs). The combination of different essential oils may amplify the free radical scavenging activity by introducing more diversity into the mixture of chemicals. In this research, we investigated the antioxidant activity of three essential oils derived from *Mentha longifolia*, *Mentha aquatica*, and *Mentha suaveolens*, individually and combined, by simplex lattice design. *In vitro* antioxidant activity was measured by DPPH, ABTS, and total antioxidant activity assays. Gas chromatography mass spectrometry analysis showed that peperitenone oxid was present at a high concentration of 40.10% and 14.36% in *M. suaveolen* and *M. longifolia*, respectively. Moreover, elemol and linalool were found at concentrations of 12.12% and 7.96%, followed by α-Terpineol at a concentration of 4.07%, in *M. aquatica*. The individual essential oils have been found to possess diverse levels of scavenging activity for DPPH, ABTS, and TAC. The optimal antioxidant mixture was found to be a mixture of 50% *M. longifolia*, 27% *M. aquatica*, and 23% *M. suaveolens*, based on the design approach, with the lowest IC_50_ values of 50.22 μg/mL for DPPH, 11.90 μg/mL for ABTS, as well as the highest total antioxidant capacity 425.01 mg AAE/mL EO, showing less than 5% deviation from predicted values, indicating high model accuracy. According to the ADMET properties, the studied essential oils exhibit advantageous pharmacokinetic profiles and good oral bioavailability. The *in silico* prediction revealed that elemol showed the highest binding affinity toward the NADPH oxidase protein, supporting their potential applications in pharmaceuticals and other bioactive formulations.

## Graphical Abstract

**Figure:**
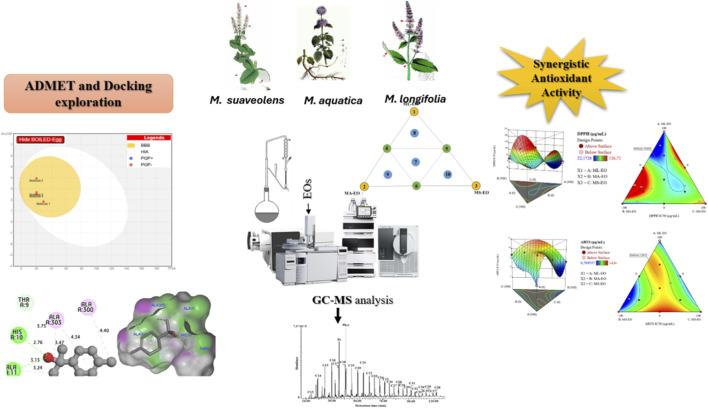

## 1 Introduction

Nowadays, growing interest in natural antioxidants reflects their potential as safe, biologically compatible compounds capable of protecting cells from damage induced by reactive chemical species. Oxidative stress occurs when there is too much production of reactive chemical species (RCS), which include reactive oxygen and nitrogen species (Reddy, 2023). These are constantly formed during normal metabolism and environmental exposure. Due to their high reactivity, these species easily interact with cellular components. This interaction disrupts redox balance and causes oxidative damage to important molecules like lipids, proteins, and DNA (Hossain et al., 2025). Such damage can affect their function and contribute to several chronic and degenerative diseases, including heart disorders, neurodegenerative diseases, inflammatory conditions, metabolic syndromes, and cancer. Nevertheless, synthetic antioxidants, such as butylated hydroxytoluene (BHT) and butylated hydroxyanisole (BHA), widely used in the food industry, could cause hepatorenal damage and promote the development of tumors (Chouikh et al., 2025). Therefore, finding effective natural sources of antioxidants that can neutralize RCS has become a key focus in both biomedical and food science research (Chibuye et al., 2024).

Essential oils (EOs) are a significant group of natural products, piquing the interest of scientists because they are composed of several volatile (aromatic) substances and have multiple biological activities (Sousa et al., 2023). EOs contain significant amounts of terpenes, terpenoids, and phenolic compounds, all of which have antioxidant properties, inhibiting the formation of peroxides by donating (hydrogen or electron) to form free radicals (Huang et al., 2025). The chemical composition of EOs is a major determiner of their functional properties, particularly the proportions of their principal bioactive chemical constituents. Several major components of EO have been documented as having substantial antioxidant and antimicrobial activity, such as menthol, menthone, thymol, carvone, α-terpinene, carvacrol, piperitenone oxide, and linalyl acetate. These constituents work via various mechanisms, including scavenging free radicals, disrupting cell membranes of microorganisms, and modulating oxidative mechanisms (Masyita et al., 2022; Sfaxi et al., 2025). Some monoterpenes including menthol, menthone or α-terpinene can have changes in membrane permeability (due to their antioxidant actions) (Hazzit et al., 2009; Khaleel et al., 2018), while some phenolic compounds like carvacrol have strong redox (anti-oxidant) and antimicrobial properties (Xu et al., 2008; Yanishlieva et al., 1999). In addition to these effects, many oxygenated monoterpenes such as linalyl acetate and piperitenone oxide show significant anti-inflammation and antimicrobial activity as well (Alqahtani et al., 2023). The direct antioxidant effects of these plant-derived oils are likely to be enhanced by the synergistic action of different oil constituents, making EOs more potent than isolated molecules in terms of their antioxidant potential (Benamar-Aissa et al., 2023). Because of their complex chemical structure, EOs represent a new way for developing natural antioxidant products.

*Mentha* (Lamiaceae) is a substantial genus of aromatic plants that has a rich history of traditional herbal medicine and a high concentration of essential oils. The most well-known members of the genus include *Mentha longifolia*, *Mentha aquatica*, and *Mentha suaveolens*, all of which have been traditionally used for their unique chemical properties and their historical use for medicinal purposes.

*Mentha longifolia* L., or Himalayan silver mint, is a medicinal plant from the Lamiaceae family, widely known in Morocco as “naana touil.” It is utilized in folk medicine across countries like Iraq, Iran, Pakistan, and Turkey, treating various conditions such as gastrointestinal and respiratory disorders, infectious and inflammatory diseases, and menstrual issues (Tourabi et al., 2023). The plant is economically significant for the food and pharmaceutical industries. Scientific research has validated its pharmacological properties, including antihemolytic, anti-inflammatory, antibacterial, hepatoprotective, anticancer, and gastroprotective effects. Additionally, essential oils extracted from *M. longifolia* have therapeutic uses, serving as powerful free radical scavengers and effective antimicrobial agents against various pathogens (Farzaei et al., 2017).

*Mentha aquatica* L., or water mint, is a perennial herbaceous plant from the Lamiaceae family and is found worldwide, excluding South America and Antarctica. It is traditionally used in countries like Vietnam, Algeria, South Africa, and Arab nations to remedy ailments such as colds, respiratory issues, coughs, and ulcerative colitis. Its applications include tonic, stimulant, digestive, and sedative effects. Scientific studies have validated its various pharmacological properties, which encompass insecticidal, antihemolytic, anti-inflammatory, antimicrobial, hepatoprotective, anticancer, gastroprotective, and antiemetic effects. The essential oil of *M. aquatica* has demonstrated strong free radical scavenging and antimicrobial activities (Pires et al., 2024; Tourabi and Baghouz, 2024).

*Mentha suaveolens* L., or Pineapple mint, is also called “Mchehtro” in Morocco. People often notice its essential oil because piperitenone oxide takes the lead, with other monoterpenes like limonene, carvone, and 1,8-cineole tagging along (El Hachlafi et al., 2023). This oil is not just pleasant to smell; it is packed with bioactive properties. In folk medicine, *M. suaveolens* is used to help with digestion, fevers, and breathing problems, plus its calming and carminative effects (Jamila et al., 2020). Modern research backs this up: it works as an antioxidant, fights microbes, and eases inflammation (El Hachlafi et al., 2023). All in all, tradition and science agree that this plant’s oil is a solid source of natural bioactive compounds.

Recent studies indicate that combining different species of essential oils (EOs) can improve their antioxidant activity as a combined treatment, compared to that reported for individual essential oils. Still, a clear understanding of these combined activities has been obscured by a lack of knowledge about the optimal ratios and mechanisms involved. For these reasons, our research aims to target three different species of the *Mentha* species, namely, *Mentha longifolia*, *Mentha aquatica*, and *Mentha suaveolens*, to chemically analyze their essential oils, as well as characterize and explore individual antioxidant abilities by mixture modeling optimization, to investigate their combined effect.

## 2 Materials and methods

### 2.1 Plant matrix and essential oil extraction

Aerial parts of *M. longifolia* and *M. suaveolens* were collected in the city of Ifran (Moroccan Middle Atlas, latitude 33°31′35″N; longitude 5°06′36''; altitude 1,648 m) in June 2025, and were identified by Professor Amina BARI, botanist from the biology department at Sidi Mohamed Ben Abdellah University. Likewise, the aerial parts of *M. aquatica* were collected in June 2021 in Merja Zerga (or Moulay Bousselham lagoon, latitude 34° 52′42.96″N, longitude −6° 17′35.99″W, altitude 12 m). The reference specimen was deposited in the faculty herbarium under the numbers 001MLAV202162 for *M. longifolia*, 002MAMZ2021 for *M. aquatica*, and 003MSMX202571 for *M. suaveolens*. Next, the harvested plants were dried in the dark for 2 weeks, then the leaves were removed, reduced to fine particles, and stored in a glass bottle in a dark place protected from heat until extraction. Then the dried leaves of *M. longifolia*, *M. aquatica*, and *M. suaveolens* (100 g) were lightly broken, the plant material was placed in a 2 L flask with 1 L of distilled water, and the mixture was heated under reflux for 3 h using a Clevenger-type apparatus (Tourabi and Baghouz, 2024). The obtained EO was separated from the water with anhydrous sodium sulfate, stored in a closed glass bottle, and kept at 4 °C in the dark until the *in vitro* test.

### 2.2 GC-MS-MS analysis of EOs

Gas chromatography technique with an ion trap mass spectrometry device was employed for GC-MS analysis (Trace GC-MS-TQ8040 NX, Shimadzu) of the essential oils of *M. longifolia*, *M. aquatica,* and *M. suaveolens* following the protocol described by (Tourabi and Baghouz, 2024), with slight modification. Regarding chromatographic separations, RTxi- 5 Sil MS capillary column (30 m × 0.25mm ID x 0.25 µm) was used. Scan range: 40–650 amu; scan rate: 3.9 scans/s; transfer line and ion source temperatures: 300 °C and 200 °C, respectively. The oven temperature was programd to increase from 40 °C to 280 °C at a rate of 5 °C/min; the injector temperature was 250 °C; helium was used as the carrier gas at a flow rate of 1 mL/min; and 1 µL of hexane essential oil solution was injected; the split ratio was 1:30. Retention times were compared to those of authentic samples, their linear retention indices were compared to the C8-C29 alkane series, and a computer comparison with mass spectra from commercial libraries (NIST version 2019) and laboratory-developed ones, consisting of pure substances and components of known oils and MS literature data, was used to determine the identification of the components.

### 2.3 Antioxidant assessment

#### 2.3.1 Assessment of DPPH radical scavenging capacity

The antioxidant potential of the extracts was determined using the DPPH free radical scavenging method, adapted from the procedure described by (Tourabi et al., 2025a). Briefly, 50 μL of the plant extract was mixed with 825 μL of a freshly prepared DPPH ethanolic solution (60 µmol) and incubated in the dark for 30 min. After incubation, the decrease in absorbance was measured at 517 nm with a UV–Vis spectrophotometer (PG Instruments Ltd., Model T60U). For the control, 50 μL of 70% ethanol was combined with 825 μL of the DPPH solution under the same conditions. The scavenging activity of the extracts against DPPH radicals was calculated according to the following Equation 1:

$$\text{Inhibition }\left(\%\right)=\left[\frac{\mathrm{AC}‐\mathrm{As}}{\mathrm{Ac}}\right]\times 100$$ (1)

where *Ac* is the absorbance of the control, and *As* i is the absorbance in the presence of the extract.

#### 2.3.2 ABTS radical scavenging capacity

Antioxidant activity was determined by the ABTS radical scavenging method according to the protocol stated by (Tourabi et al., 2025b). It is based on the reduction of the ABTS•^+^ radical (2,2′-azino-bis(3-ethylbenzothiazoline-6-sulfonic acid). Briefly, a final volume of 875 μL of freshly prepared ABTS diammonium salt solution (7 mM) was mixed with 50 μL of the tested samples. The reaction mixture was incubated in the dark conditions for 30 min. Absorbance was then measured at 734 nm in a PerkinElmer Lambda 40 UV–Visible spectrophotometer against an ethanol blank. The antioxidant capacity was expressed as % inhibition of ABTS radical, and expressed as IC_50_ (µg/mL) ± SD, with trolox as a standard.

#### 2.3.3 Total antioxidant capacity (TAC)

Total antioxidant capacity (TAC) was estimated by the phosphomolybdenum method (Tourabi and Baghouz, 2024). According to this, 100 μL samples were mixed with 1 mL of a reagent solution that contained sulfuric acid (0.6 M), Sodium phosphate (28 mM), and ammonium molybdate (4 mM). These mixtures were then heated to 95 °C for a period of 90 min to ensure complete development of color. Upon completion of heating, these mixtures cooled down to room temperature, and then absorbance was measured at a wavelength of 700 nm by a PerkinElmer Lambda 40 UV/VIS spectrophotometer, using ethanol as a blank. Antioxidant activity was measured as milligrams of ascorbic acid equivalent per gram dry weight (mg AAE/g DW) by generating a calibration curve by preparing a standard curve using ascorbic acid.

### 2.4 Experimental design

#### 2.4.1 Mixture design

In this study, an augmented simplex-lattice design experiment was employed to determine the optimal combination of essential oils (EOs) from *M. longifolia*, *M. aquatica*, and *M. suaveolens* according to the methodology presented by Akmal et al. (2023). The composition of the mixtures of EOs is given in Table 1. In this mixture design, individual EOs were varied from 0 to 1, and the total of the three EOs was fixed to be 1. The antioxidant capacity of the mixtures of EOs was evaluated using DPPH_IC50_, ABTS_IC50_, and total antioxidant capacity.

**TABLE 1:** Mixture formulation variables.

Compenents	Coded variables	Level -	Level +
ML-EO	M_1_	0	1
MA-EO	M_2_	0	1
MS-EO	M_3_	0	1
Total of proportions	​	1	​

#### 2.4.2 Experimental matrix and mathematical model

**FIGURE 1:**
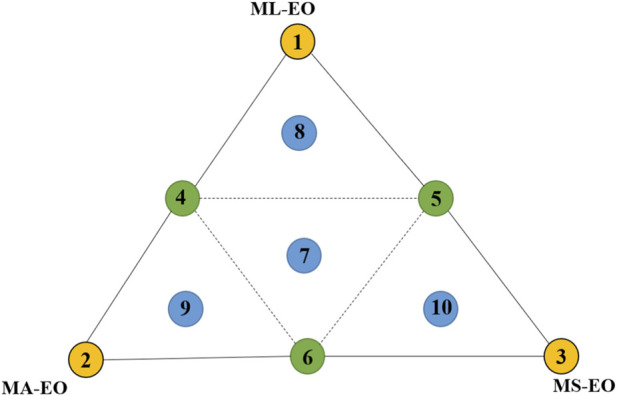
Equilateral triangle representing the arrangement of molecular mixtures using the simplex-Lattice design method (ML-EO, MA-EO, MS-EO).

This experiment consists of mapping 12 experimental points to an equilateral triangle (Figure 1), symbolically representing the proportions of the varied components. The three pure components lie on the vertices of the equilateral triangle (M_1_, M_2_, and M_3_). Binary mixtures involving the combination of the other two components in an equal ratio (0.5/0.5) lie on the midpoints of the edges of the equilateral triangle (M_4_, M_5_, and M_6_). Points representing the mixture of all three molecules with an equal ratio (0.33/0.33/0.33) lie on the centroid of the equilateral triangle (M_7_). To make the results reliable and consistent, the experiment will be performed thrice with control points lying on the ternary mixture with varied ratios (0.66/0.17/0.17) at points M_10_, M_11_, and M_12_.

A cubic polynomial model was used to fit the system responses to the selected variables. This was useful to investigate the combined effects and the interactions between the variables, and the system responses are determined by Equation 2.

$$Y=\beta 1\;M_{1}+\beta 2\;M_{2}+\beta 3\;M_{3}+\beta 12\;M_{1}M_{2}+\beta 13\;M_{1}M_{3}+\beta 23\;M_{2}M_{3}+\beta 123\;M_{1}M_{2}M_{3}$$ (2)

In the equation, Y is representative of the experimental IC_50_ value (μg/mL) and TAC value (mg AAE/mL EO). Coefficients M_1_, M_2_, and M_3_ are indicative of the linear effects of each component tested separately. While interaction effects stemming from combinations of essential oils are represented by coefficients M_12_, M_13_, and M_23_. M_123_ is representative of interaction effects in the ternary combination. This model equation is useful for evaluating quantitatively the individual and interaction effects of various components with respect to the response quantitatively.

### 2.5 *In silico* pharmacokinetic and drug-likeness predictions

The pharmacokinetic and drug-likeness properties of the major compounds extracted from *Mentha longifolia*, *Mentha aquatica*, and *Mentha suaveolens* essential oils were evaluated using computational prediction tools. Key physicochemical descriptors such as molecular weight, lipophilicity, polarity, hydrogen bonding capacity, and flexibility were calculated (Fadili et al., 2022).

Drug-likeness was assessed based on Lipinski rules. Gastrointestinal absorption and blood-brain barrier (BBB) permeability were predicted (Ed-Dahmani et al., 2024; El Fadili et al., 2025), together with general ADME behavior (Seddoqi et al., 2024). The BOILED-Egg model was used to classify compounds according to their probability of intestinal absorption and brain penetration, including P-glycoprotein substrate prediction (Ed-Dahmani et al., 2024). In addition, Egan’s model and bioavailability radar were used to evaluate whether the molecules fall within the optimal drug-like chemical space (El Fadili et al., 2026).

#### 2.5.1 Molecular docking study

The 3D structure of NADPH oxidase, selected as the antioxidant-related target protein, was retrieved from the Protein Data Bank and prepared by removing water molecules and any co-crystallized ligands. Hydrogen atoms were added, and the structure was minimized to ensure stability before docking (Ed-Dahmani et al., 2025; El Fadili et al., 2023b).

The major compounds identified from essential oils, namely, piperitenone oxide (M1), elemol (M2), linalool (M3), and α-Terpineol (M4), were built and optimized using standard molecular modeling procedures. Molecular docking was performed within the active site of NADPH oxidase to explore the binding behavior of each compound. The best docking poses were selected according to binding affinity and interaction patterns. The resulting complexes were analyzed to identify key interactions contributing to protein-ligand stability.

### 2.6 Statistical analysis

ANOVA was used to assess the appropriateness of the model. An F-test, calculated by the ratio of the mean square regression (MSR) and the mean square residual (MSres), was used to determine whether or not the model was statistically significant at 95% (p < 0.05). To assess whether this model fits well, the lack-of-fit has been tested by comparing the MSLOF (mean square lack of fit) with the MSPE (mean square pure error). If the *p*-value is greater than 0.05, then the lack-of-fit is not significant, and thus, the model does indeed fit the experimental data well. The quality of the model was assessed using the coefficient of determination (*R* ^2^), along with the adjusted *R* ^2^ values, which provide a more reliable estimation of model performance. All statistical analysis for the study was conducted using the Design- Expert software version 13. Experimental data were reported as the mean ± standard deviation (SD) of three replicates (n = 3). To optimize, contour plots and 3D response surface plots were made to assess the interaction of the mixture components.

## 3 Results and discussion

### 3.1 Chemical profile of three EOs

Table 2 shows the chemical composition, relative percentages, and extraction yields for the three essential oils extracted from *M. longifolia*, *M. aquatica,* and *M. suaveolens*. The extraction yields were 1.33% (*v/w)* for *M. longifolia*, 0.58% (*v/w)* for *M. aquatica*, and 1.19% (*v/w)* for *M. suaveolens*. Each oil had its own phytochemical profile, while *M. longifolia* had 13 compounds, *M. aquatica* had 26, and *M. suaveolens* had 22. The main phytochemicals found were oxygenated monoterpenes and sesquiterpenes. Some compounds appeared in all three oils, while others were unique to each, showing chemical differences among the species and helping explain their different therapeutic effects. These unique chemical profiles may also be linked to their various pharmacological activities, such as antimicrobial, antioxidant, anti-inflammatory, and analgesic effects (Banjari et al., 2020; Kim et al., 2020; Zengin and Baysal, 2014).

**TABLE 2:** Phytochemical constituents of essential oils extracted from *Mentha longifolia*, *Mentha aquatica*, and *Mentha suaveolens*.

Compounds*	Area %			Linear retention intex (RI)	
	ML-EO	MA-EO	MS-EO	RI_Cal**_	RI_Lit***_
1-Octen-3-ol	-	-	0.41	969	994
1,8-Cineole	0.47	0.32	0.44	1,059	1,031
Linalool	0.44	**7.96**	-	1,082	1,082
p-Menthan-4-ol	-	-	1.20	1,127	1,162
(−)-Terpinen-4-ol	-	-	0.66	1,137	1,177
Borneol	-	1.38	0.45	1,138	1,148
α-Terpineol	-	**4.07**	-	1,143	1,163
Trans-linalool oxide	-	0.70	-	1,162	1,072
Estragole	-	-	0.31	1,172	1,177
1,2-Epoxylinalool	-	2.00	-	1,182	1,061
(+)-carvone oxide	**2.07**	-	0.38	1,202	1,251
2-Hydroxy-1,8-cineole	-	0.30	-	1,247	1,219
trans-pyranoid linalool oxide	-	0.56	-	1,255	1,175
Trans-ascaridol glycol	-	-	0.33	1,259	1,268
*α*-terpinene	-	-	**1.46**	1,259	1,205
Carvacrol	0.39	-	-	1,262	1,297
Piperitenone oxide	**14.36**	0.53	**40.10**	1,236	1,366
Linalyl acetate	-	**4.78**	-	1,272	1,258
1-Butanone, 1-bicyclo [4.1.0]hept-7-yl-	16.11	-	-	1,273	-
2,3-Pinanediol	-	0.80	-	1,276	1,244
*p*-Cymen-7-ol	-	-	0.73	1,284	1,287
8,9-Dehydrothymol	-	-	1.52	1,293	1,221
8-Hydroxylinalool	-	0.57	-	1,325	1,333
p-Mentha-1,8-diol	-	0.45	​	1,331	1,326
6-Hydroxycarvotanacetone	0.48	-	-	1,346	1,395
Geranyl acetate	-	1.16	​	1,352	1,380
exo-2-Hydroxycineole acetate	-	0.30	-	1,386	1,333
Patchouli alcohol	-	0.53	-	1,420	1,639
Cinerolone	**1.43**	-	**2.63**	1,426	1,405
*p*-Menthane-1,2,4-triol	**1.74**	-	0.51	1,503	1,470
Elemol	-	**12.12**	-	1,522	1,549
1,13-Tetradecadien-3-one	-	-	0.40	1,529	​
(−)-Globulol	**1.69**	-	-	1,530	1,583
Viridiflorol	-	3.44	**1.50**	1,530	1,590
(−)-Spathulenol	0.62	-	0.75	1,536	1,574
(+)-ledol	-	-	0.52	1,539	1,602
cis-14-Nor-Muurol-5-en-4-one	-	0.41	0.68	1,540	1,688
τ -cadinol	-	1.41	0.57	1,580	1,640
α-Cadinol	0.49	​	**1.24**	1,580	1,654
Eudesm-4 (14)-en-11-ol	-	**5.73**	-	1,593	1,484
γ -eudesmole	-	1.05	-	1,626	1,626
Diethyl phthalate	41.73	31.89	17	1,639	1,610
Nerolidol-epoxyacetate	-	0.29	-	1,687	1,687
8.alpha.,11-elemadiol	-	0.42	-	1710	1734
Eudesm-4-en-3-one	-	2.71	-	1798	1745
Chemical class					
OM	21.06	25.88	48.95	​	
OS	2.8	28.11	5.26	​	
MH	-	-	1.46	​	
O	57.84	31.89	18.12	​	

* Components were identified by mass spectrometry (MS) and retention index (RI).** The Kovats index was obtained by using an alkane series (C8-C29) with a MS, capillary column. *** Kovats indices (retention indices) were obtained from data libraries. The chemical composition of the essential oils’ constituents can be classified into four groups: OM, oxygenated monoterpenes; OS, oxygenated sesquiterpenes; MH, monoterpene hydrocarbons; and O, others. The key components are presented in bold for clarity.

In *M. longifolia* essential oil (ML-EO), the major group of compounds is found to be oxygenated monoterpenes. Among the identified components of the essential oil, piperitenone oxide (14.36%) was found to be the major compound. This compound makes a major contribution to the biological activities of the essential oil, such as antimicrobial, antioxidant, insecticidal, and anti-inflammatory properties. In addition to this compound, other components of the oxygenated monoterpenes have also been identified to be present in moderate amounts, such as cinerolone (1.43%), p-Menthane-1,2,4-triol (1.74%), and (+)-carvone oxide (2.07%), which have a major contribution to the aromatic value of the essential oil.

The EO of *M. aquatica* has been studied and has been shown to contain oxygenated monoterpenes and sesquiterpenic compounds primarily. It has identified compounds including elemol (12.12%), linalool (7.96%), and eudesm-4 (14)-en-11-ol (5.73%), linalyl acetate (4.78%), and α-terpineol (4.07%). Linalool is an oxygenated monoterpene, which has been reported in the literature to possess antibacterial, insecticidal, and antioxidant properties (Tourabi and Baghouz, 2024). In comparison, in the study of Ferrati et al., the compound linalool was again identified as a main compound of the essential oil of *M. aquatica*, thus validating the consistent appearance of the compound in the essential oils of the plant (Ferrati et al., 2023). Furthermore, linalyl acetate, which is also commonly reported, possesses both antimicrobial and relaxing properties. Moreover, linalyl acetate has also been frequently found co-occurring with linalool in aromatic plants.

Furthermore, α-terpineol is an additional chemical that has been documented to possess biological activity and may contribute to the activity of *M. aquatica* essential oils, particularly with respect to antibacterial and antifungal activity (Khaleel et al., 2018). Additionally, the presence of the sesquiterpenoid elemol and eudesm-4 (14)en-11-ol has demonstrated biological activities, including antioxidant, antimicrobial, and antiproliferative properties (Bruna et al., 2022).

The main compound in *M. suaveloens* (MS-EO) was identified as a monoterpenic ketone named peperitenone oxide (40.10%), with minor constituents being identified as cinerolone (2.63%), viridiflorol (1.50%), and α-cadinol (1.24%), all of which indicate its antimicrobial and anti-inflammatory potential (Božović et al., 2015). Cinerolone, viridiflorol, and α-cadinol are considered sesquiterpenes that may support the antioxidant and antimicrobial activity of *M. suaveloens* EO and also provide additional aromatic characteristics (Vambe et al., 2025). Consequently, this may support both the therapeutic and the aromatic characteristics of the MS-EO. Previous research on *Mentha* spp. Supports these conclusions, while peperitenone derivatives are identified as the major group of bioactive compounds implicated in altering the pharmacological characteristics of the group.

The studied essential oils (EOs) extracted from *M. longifolia*, *M. aquatica*, and *M. suaveolens* were primarily characterized by the presence of oxygenated monoterpenes. However, they each have a specific chemical composition. The main component of ML-EO is piperitenone oxide (14.36%), while MA-EO contains elemol (12.12%) and linalool (7.96%), and MS-EO exhibits relatively high levels of piperitenone oxide (40.10%). Cinerolone, α-cadinol, and linalyl acetate were present in small amounts and are responsible for the antimicrobial, antioxidant, and aromatic activity of the EOs. These variations may be related to several factors such as geographical origin, environmental conditions, harvesting period, and extraction methods, which are known to influence the chemical composition of essential oils.

### 3.2 Antioxidant potential of selected EO_S_

**FIGURE 2:**
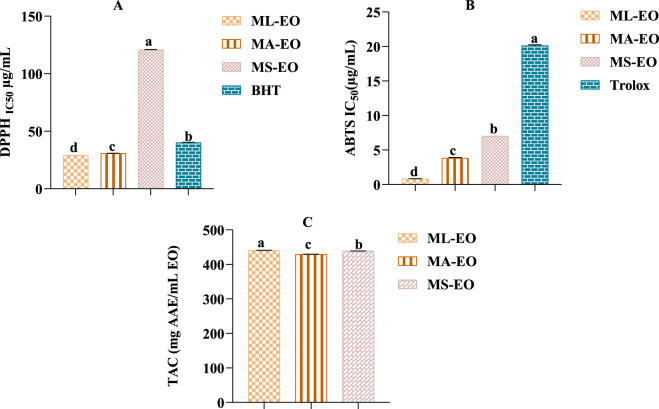
Antioxidant potential of three essential oils using **(A)** DPPH assay, **(B)** ABTS assay, and **(C)** TAC assay. DPPH: 2,2-Diphenyl-1-picrylhydrazyl test; ABTS: 2,2′-azino-bis (3-ethylbenzothiazoline-6-sulfonic acid) assay; TAC, Total antioxidant capacity; BHT, butylated hydroxytoluene. Results are indicated as mean ± SD and represented for three independent replicates. Significance is indicated by different letters between groups. Significance was agreed upon at a level of p < 0.05.

Figure 2 represents the antioxidant activity of three essential oils (ML-EO, MA-EO, and MS-EO), which were tested using three different *in vitro* analysis techniques: DPPH radical scavenging activity, ABTS radical cation decolorization assay, and total antioxidant activity (TAC). Commercial antioxidants (BHT and Trolox) were used as standards. Different lowercase letters on top of the bars show significantly different samples (p < 0.05).

The DPPH assay (Figure 2A) is a measure of the capacity of these compounds to neutralize the DPPH• radicals, giving information about their free radical-scavenging ability. Among these essential oils, it has been found that the ML-EO has the lowest IC_50_ value and therefore, it is most efficient in free radical scavenging activity with an IC_50_ value of 29.17 ± 0.01 μg/mL, followed by MA-EO with an IC_50_ value of 30.64 ± 0.22 μg/mL, better than BHT, while on the other hand, MS-EO is less efficient in DPPH scavenging capacity with the highest IC_50_ value of 120.86 ± 0.12 μg/mL. The above-obtained results have been confirmed by previous studies, which confirm that both ML-EO and MA-EO possess the highest antioxidant activity due to their richness with various antioxidant molecules like monoterpenoids and sesquiterpenoids (Tourabi and Baghouz, 2024; Tourabi et al., 2023).

In the ABTS test, the assay is based on the formation of a blue-green ABTS•^+^ radical, which is reduced by the test samples through electron or hydrogen transfer. A similar finding was also noted in the ABTS assay (Figure 2B). In these tests, ML-EO showed the highest antioxidant activity among the examined essential oils with an IC_50_ value of 0.81 ± 0.03 μg/mL. Therefore, MA-EO showed moderate activity with an IC_50_ value of 3.82 ± 0.10 μg/mL, and the weakest ABTS radical scavenging activity was exhibited by MS-EO with a display of an IC_50_ of 7.02 ± 0.00 μg/mL. As it turned out, trolox showed moderate antioxidant activity with an IC_50_ of 20.12 ± 0.29. However, it is important to note that the essential oil showed antioxidant activity that surpassed those of commonly used standards, thus portraying its highest antioxidant activity. The obtained data plainly showed that there is a notable relationship between the antioxidant activity of molecules and their molecular structures, because electron-donating groups increase antioxidant activity through enhanced redox reactions.

In the phosphomolybdenum TAC assay (Figure 2C), antioxidants reduce Mo(VI) to Mo(V), which under acidic conditions forms a green phosphate/Mo(V) complex. Results showed a slightly different pattern between the tested samples. ML-EO had the highest TAC value of 440.72 ± 0.30 mg AAE/mL EO, followed by MS-EO with 438.80 ± 0.31 mg AAE/mL EO, whereas the lower capacity was noticed with MA-EO, with a corresponding value of 428.99 ± 0.94 mg AAE/mL EO. The obtained results suggest that the essential oils under study could be rich in components that are highly electron-donating and hence more appropriately detected by this test.

Overall, the results indicate that ML-EO has the most stable and strongest radical-scavenging ability compared to the other two essential oils. While MA-EO and MS-EO have a lower antioxidant ability, this might be attributed to the lower effect of such functional groups. All these differences might be attributed to the variation in chemical composition, such as phenolic, terpenoids, and oxygenated monoterpenes (Sharopov et al., 2015). In addition, the antioxidant activity observed in ML-EO is likely due to the presence of a majority of the monoterpenoids, which contain a large number of oxygen-derived products. The primary monoterpene is piperitenone oxide, as well as other compounds, including cinerolone, p-menthane-1,2,4-triol (+)- carvone oxide, which have also been demonstrated to exhibit antioxidant activity. The antioxidant activity displayed by these compounds is thought to occur predominantly through free radical scavenging and donating hydrogen atoms to those involved in the oxidation chain reaction (Božović et al., 2015; Pombal et al., 2020). Linalool, linalyl acetate, and α-terpineol are the major oxygenated monoterpenes identified in MA-EO, which have antioxidant activity through ROS scavenging and lipid peroxidation prevention (Bicas et al., 2011; dos Santos et al., 2022). There can additionally be an enhancement of antioxidant activity when sesquiterpene components (e.g., elmol, eudesm-4 (14)-en-11-ol) are also present, providing for potential synergistic enhancement of compounds (Khan, 2008). Similarly, in MS-EO, the predominant piperitenone oxide, along with viridiflorol and α-cadinol, provides evidence of the oil’s ability to act as an antioxidant, because of the way those sesquiterpenes are known to function in providing electron-donating capacity and stabilization of free radicals (Božović et al., 2015; Trevizan et al., 2016). Therefore, the essential oils’ ability to function as antioxidants can be directly related to the various major constituents identified within the oil, which display antioxidant properties either in and of themselves or when used in combination with one another through radical scavenging, hydrogen donating and inhibiting oxidative processes, as described in current literature.

Overall, the results indicate that all three essential oils have strong antioxidant activities; however, the potency differs according to the antioxidant mechanism used in the experiments. Also, the fact that there are differences among the assays conducted illustrates the effectiveness of conducting multiple assays when determining the antioxidant properties of any compound, since different assays investigate different mechanisms of antioxidants.

### 3.3 Simplex lattice design

Table 3 shows the Simplex lattice mixture design with three principal EOs, namely, ML-EO, MA-EO, and MS-EO, respectively, and their respective antioxidant activities determined by DPPH and ABTS assays and TAC. These essential oils have been widely recognized for their several biological properties. The study of their mixture effects can therefore help in designing a product or mixture capable of resisting oxidative stress with additional benefits. To our knowledge, there have not been any studies conducted before on the composite antioxidant effects of *M. longifolia*, *M. aquatica*, and *M. suviolens* using this experimental protocol. In this design, a total of 12 experimental mixtures, including pure oils, binary, and ternary combinations, were measured, where each value corresponds to the mean ± SD of three independent replicates. The antioxidant activity showed a considerable variation based on the composition of the mixtures. The DPPH concentration ranged from 29.17 ± 0.01 to 126.72 ± 0.12 μg/mL, and ABTS ranged from 0.81 ± 0.03 to 14.64 ± 0.31 μg/mL. TAC concentrations were more variable, ranging from 356.35 ± 1.56 to 829.80 ± 0.31 mg EAA/mL EO. Among all mixtures tested, mixture 5, with a combination of ML-EO and MS-EO in a ratio of 0.5:0.5, recorded the highest TAC value, indicating a very strong combined antioxidant activity. Mixture 1, with a composition of only ML-EO, and mixture 10, with a composition of ML-EO, MA-EO, and MS-EO in a ratio of 0.67, 0.17, and 0.17, respectively, recorded the highest DPPH radical scavenging capacity of 29.17 ± 0.01 and 42.77 ± 0.18 μg/mL, respectively. The results obtained using the ABTS test showed that mixture 11, with a composition of ML-EO, MA-EO, and MS-EO in a ratio of 0.17, 0.67, and 0.17, respectively, recorded a higher radical neutralization capacity with an IC_50_ value of 5.96 ± 0.09 μg/mL compared to other controls, trolox, which recorded a higher IC_50_ value of 20.12 ± 0.29 μg/mL.

**TABLE 3:** Simplex lattice design matrix and corresponding results for the antioxidant activity of essential oil mixtures.

​	​	​	Observed responses			
Experiment number	ML-EO	MA-EO	MS-EO	DPPH (µg/mL)	ABTS (µg/mL)	TAC (mg EAA/mL EO)
1	1	0	0	29.17 ± 0.01	0.81 ± 0.03	440.72 ± 0.30
2	0	1	0	30.64 ± 0.22	3.82 ± 0.10	428.99 ± 0.94
3	0	0	1	120.86 ± 0.12	7.02 ± 0.00	438.80 ± 0.31
4	0.5	0.5	0	122.52 ± 0.16	6.09 ± 0.02	712.21 ± 4.56
5	0.5	0	0.5	117.31 ± 0.17	3.29 ± 0.00	829.80 ± 0.31
6	0	0.5	0.5	63.54 ± 0.20	14.64 ± 0.31	666.54 ± 0.35
7	0.33	0.33	0.33	52.77 ± 0.51	11.22 ± 0.73	534.98 ± 2.18
8	0.33	0.33	0.33	52.78 ± 0.50	10.22 ± 0.18	574.98 ± 0.62
9	0.33	0.33	0.33	50.97 ± 0.55	6.14 ± 011	554.89 ± 0.23
10	0.67	0.17	0.17	42.77 ± 0.18	11.82 ± 0.40	356.35 ± 1.56
11	0.17	0.67	0.17	126.72 ± 0.12	5.96 ± 0.09	682.35 ± 0.30
12	0.17	0.17	0.67	55.17 ± 0.10	9.45 ± 0.11	698.40 ± 0.66
BHT	​	​	​	40 ± 0.11	-	-
Trolox	​	​	​	-	20.12 ± 0.29	-

Experiments were performed in three independent replicates, and values are presented as means ± standard deviation.

### 3.4 Statistical verification of the proposed model

Analysis of variance for the effects of mixture components on antioxidant activity is indicated in Table 4. The p-values (p < 0.0001) were below 0.05 and hence statistically significant for all responses-DPPH_IC50_, ABTS_IC50_, and TAC. This significance was further confirmed by high F-values obtained for each response (168.53 for DPPH_IC50_, 1,048.16 for ABTS_IC50_, and 201.37 for TAC), which were well above the critical F-values at a confidence level of 95%, showing that the mixture components highly affected antioxidant responses.

**TABLE 4:** Analysis of variance for the three fitted models.

Response	Model	DF	SS	MS	F	*p*-value
DPPH_IC50_	R	8	24392.41	3049.05	168.53	<0.0001*
	r	5	90.46	18.09	​	​
	Lack of fit	1	40.96	40.96	3.31	0.1430
	Total	13	24,482.87	​	​	​
	*R* ^2^	​	​	0.99	​	​
	R^2^Adjusted	​	​	0.99	​	​
ABTS_IC50_	R	8	218.53	27.32	1048.16	<0.0001*
	r	5	0.1303	0.0261	​	​
	Lack of fit	1	0.0451	0.0451	2.12	0.2192
	Total	13	218.66	​	​	​
	*R* ^2^	​	​	0.99	​	​
	R^2^Adjusted	​	​	0.99	​	​
TAC	R	8	3.205 E+05	40,059.88	201.37	<0.0001*
	r	5	994.67	198.93	​	​
	Lack of fit	1	82.45	82.45	0.3615	0.5801
	Total	13	3.215^E^+05	​	​	​
	*R* ^2^	​	​	0.99	​	​
	R^2^Adjusted	​	​	0.99	​	​

R, regression; R^2^, coeficcient of determination; r, residual; DF, degre of freedom; SS, sum of squares; MS, mean square; *Statistically significant at *p* < 0.05.

**FIGURE 3:**
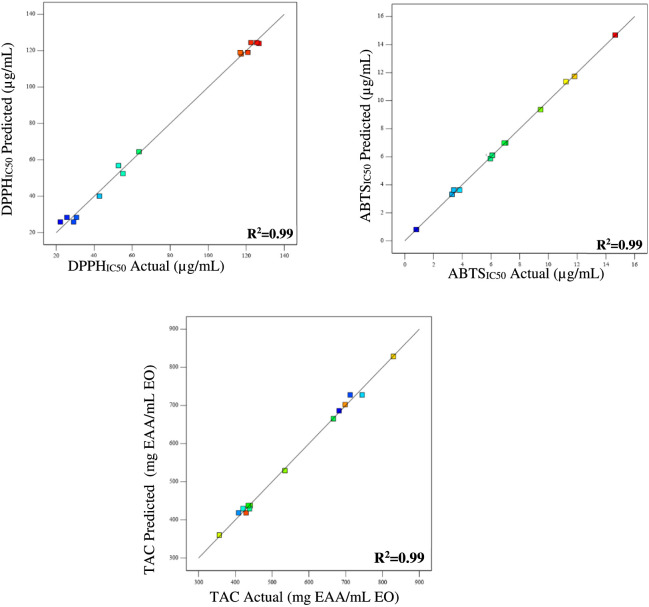
Comparison of experimental and expected values for DPPH_IC50,_ ABTS_IC50,_ and TAC, shown by the black curve.

Furthermore, the results obtained from the ANOVA lack-of-fit tests proved the validity of the obtained models, since the p-values were all above 0.05 for all responses (0.1430 for DPPH_IC50_, 0.2192 for ABTS_IC50_, and 0.5801 for TAC), with no significance in lack of fit. Additionally, it can be concluded that the calculated F-ratios for lack of fit/pure error were less than the critical F-values at a confidence level of 95% for all responses, which further assures a non-significant lack-of-fit error in all obtained models. Additionally, a high value for the coefficient of determination for all responses of 0.99 shows a good fit between experimental and predicted responses, which corresponds with linear relationships noted in estimated responses (Figure 3).

### 3.5 Components, effects, and adjusted models

Regression coefficients of the proposed models are demonstrated in Table 5. Significant correlations among variables in the mixture and antioxidant properties (DPPH IC_50_, ABTS IC_50_, and TAC) were established using regression analysis, with most regression coefficients exhibiting a significance level of less than 0.05. The regression analysis for DPPH_IC50_ response, the individual component coefficients (β1,β2, β3), and the ternary interaction term (β123) were all highly significant (p < 0.0001), showing individual oil effects and their combination effects in a significant manner on DPPH radical scavenging activity. The binary interaction terms β12 and β23 were non-significant (p > 0.05), but β13 demonstrated significance (p = 0.0002), which indicated that not all binary oil combinations have equal effects on DPPH_IC50_ response. For the ABTS_IC50_ model, individual components and most of the interaction terms, such as binary interaction terms (β12, β23) and ternary interaction terms (β123), were all significant (p < 0.05). Such observations indicate a different level of complexity in ABTS_IC50_ radical scavenging, where individual oil components and their combinations have a major role.

**TABLE 5:** Coefficients of the three proposed models with their significance levels (p-values).

Compentent	Coffecient	DPPH		ABTS		TAC	
		Estimation	*p*-value	Estimation	*p*-value	Estimation	*p*-value
A-ML-EO	β_1_	25.90	< **0.0001** *	0.80	**< 0.0001** *	437.60	0.3980
B-MA-EO	β_2_	28.37	**< 0.0001** *	3.63	**< 0.0001** *	418.60	0.3980
C-MS-EO	β_3_	119.08	**< 0.0001** *	6.98	**< 0.0001** *	429.46	0.3980
A*B	β_12_	389.34	**< 0.0001** *	15.56	**< 0.0001** *	1,199.07	**< 0.0001** *
A*C	β_13_	182.91	**0.0002** *	−2.29	**0.0242** *	1,579.94	**< 0.0001** *
B*C	β_23_	−37.14	0.1071	37.49	**< 0.0001** *	964.91	**< 0.0001** *
A*B*C	β_123_	−1,630.92	< **0.0001** *	51.52	**0.0001** *	−8,514.01	**< 0.0001** *

* Values in bold indicate statistical significance at *p* < 0.05.

Regarding the TAC response, both binary and ternary interaction terms were highly significant, with a p-value less than 0.05 (p < 0.0001), and the effects due to individual components were not significant, with a p-value of 0.3980.

To be specific, Equation 3 shows mathematical models in which the response is a function of the studied constituents after removing any non-significant coefficients from hypothesized mathematical models.

$$Y_{\text{DPPH}\;\text{IC}50}=25.90\;M_{1}+28.37\;M_{2}+119.08\;M_{3}+389.34\;M_{1}M_{2}+182.91\;M_{1}M_{3}\;‐1630.92\;M_{1}M_{2}M_{3}$$ (3)

Regarding ABTS_IC50_, the model indicated that β1, β2, β12, β23, and β123 were significance terms. These findings support the proposition that main effects β_1_, representing treatment *M. longifolia*, and β_2_, representing treatment *M. aquatica*, as well as two-factor and three-factor interaction effects, primarily with β_3_ representing treatment *M. suaveolens,* contribute greatly to establishing antioxidant activity against ABTS radicals. Equation 4 thus gives the accepted mathematical model:

$$Y_{\text{ABTS}\;\text{IC}50}=0.8071\;M_{1}+3.63\;M_{2}+6.98\;M_{3}+15.56\;M_{1}M_{2}+37.49\;M_{2}M_{3}+51.52\;M_{1}M_{2}M_{3}$$ (4)

Concerning TAC, the model indicated that β12, β13, β23, and β123 were significant terms. These outcomes support that the binary effect has a major influence on total antioxidant capacity. Consequently, Equation 5 expresses the validated mathematical model.

$$Y_{\text{TAC}}=1199.07\;M_{1}M_{2}+1579.94\;M_{1}M_{3}+964.91\;M_{2}M_{3}\;‐\;8514.01\;M_{1}M_{2}M_{3}$$ (5)

### 3.6 Mixture profile

**FIGURE 4:**
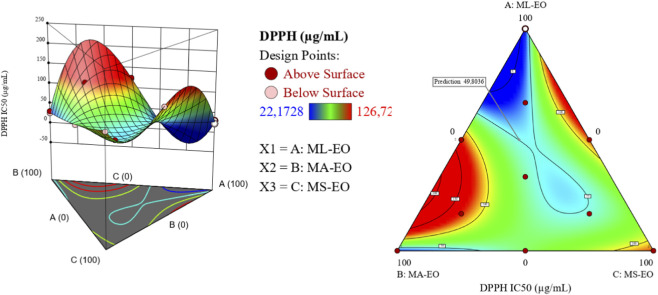
2D and 3D mixture plots of the desired compromise area, resulting in the best value of DPPH _IC50_.

**FIGURE 5:**
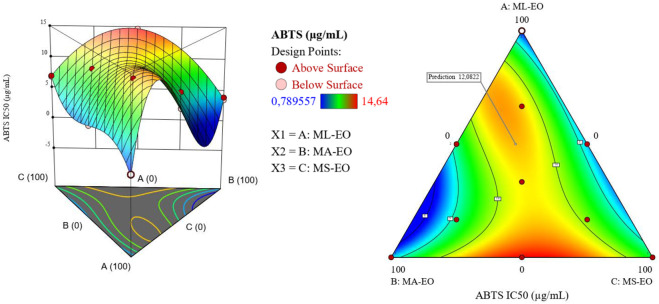
2D and 3D mixture plots of the desired compromise area, resulting in the best value of ABTS _IC50_.

**FIGURE 6:**
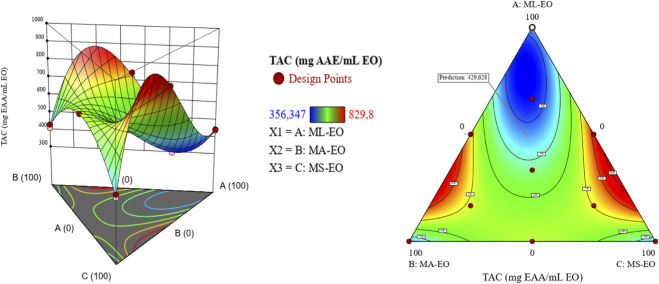
2D and 3D mixture plots of the desired compromise area, resulting in the best value of TAC.

The profile and 3D plots given in Figures 4–6 above clearly demonstrate how the varying concentrations of *M. longifolia*, *M. aquatica*, and *M. suaveolens* essential oils interact and affect the antioxidant activities (DPPH_IC50_, ABTS_IC50_, and TAC) for a given percentage concentration. These graphical diagrams enable visualization of the impact of variations in the ratios of each oil on the measured antioxidant activities and support in establishing the compositions of mixtures that result in desirable responses. The plots created using Design-Expert software were made possible through the use of iso-response curves that offer excellent assistance in identifying the most desirable conditions for formulation. In terms of the colors used in the graphical representation of the scales, the blue regions represent low IC_50_ values and high antioxidant activity, while the transition from yellow to dark red shows high IC_50_ values and low activity in both DPPH and ABTS assay results, inversely for TAC.

#### 3.6.1 Optimization of DPPH_IC50_ model

The provided 2D and 3D mixture plots (Figure 4) clearly show that the antioxidant response concentration is significantly influenced by the combination of the three key essential oils: ML-EO, MA-EO, and MS-EO. The interplay of the three factors in the three-dimensional surface plot indicates that the effects are non-linear and that the antioxidant effect is influenced not just by one essential oil alone but also by their interaction effects. The color gradient in the surface and contour plots transitions from lower DPPH_IC50_ values (blue regions) to higher values (yellow and red regions). The optimal compromise zone, indicated by the least concentration of DPPH_IC50_ (greatest antioxidant capacity), is located in the blue region of the ternary plot. The target region of the mixture design shows the lowest DPPH_IC50_ value of about 22.17 μg/mL, indicating its greatest antioxidant capability among the ternary mixture of ML-EO, MA-EO, and MS-EO. The model would additionally provide an IC_50_ of 49.80 μg/mL for another mixture formulation that exhibits high antioxidant activity relative to the optimal compromise zone.

#### 3.6.2 Optimization of the ABTS_IC50_ model

As shown in 2D and 3D surface contour plots (Figure 5), the best ABTS_IC50_ is linked to a ternary mixture of ML-EO, MA-EO, and MS-EO. It can be seen that the dark blue area represents the optimal compromise point that provides an optimal ABTS_IC50_ value of around 0.78 μg/mL for the ternary combination of ML-EO, MA-EO, and MS-EO. In addition, it can be noted that for this particular ternary combination, there is a predicted IC_50_ value of 12.08 μg/mL, denoting another possible formulation exhibiting the strongest antioxidant activity compared to that of the optimal mixture.

#### 3.6.3 Optimization of the TAC model

The mixture plots displayed in Figure 6 demonstrate that the region of highest Total Antioxidant Capacity (TAC) is visible in the red areas of both the 2D and 3D surface plots. The plots identify a maximized TAC in the red region, with values reaching up to 829.8 mg AAE/mL EO, which is considerably higher than in other areas of blue, green, and yellow regions. An optimum TAC value of about 429.62 mg AA/mL EO can be expected in the green region of the ternary plot, signifying a harmonious blend capable of achieving good antioxidant capacity through synergistic interactions. The optimum zone is identified as a compromise formulation useful for improved bioactivity.

In the same context, the use of mixture design methods has increasingly been adopted in a variety of fields of study, specifically in designing and optimizing blends of essential oils. Crespo et al. examined the synergistic antioxidant effect of a mixture of essential oils, namely, *Ocimum basilicum L*., *Origanum majorana* L., and *R. officinalis* L., using a simplex lattice mixture design to model and optimize the combined effect on DPPH antioxidant responses (Baj et al., 2018). Similarly, Elbouzidi et al. apply the same approach to identify the greatest ratios of *L. dentata*, *R. officinalis*, and *Myrtus communis* to improve antioxidant activity. The optimum combination of antioxidants for DPPH_IC50_ was found to be 20% of *Lavandula dentata,* 50% of *Rosmarinus officinalis*, and 30% of *M. communis*. Whereas the combination showing maximum ABTS_IC50_ radical scavenging activity was determined to be 18% of *L. dentata*, 43% of *R. officinalis*, and 40% of *M. communis* (Elbouzidi et al., 2024).

### 3.7 Experimental confirmation of the assumed model

Table 6 presents a comprehensive verification test for cubic models employed for calculating the antioxidant activity of a combination of EOs originating from *M. longifolia*, *M. aquatica,* and *M. suaveolens*. Such a test is crucial for validating the accuracy of the models for predicting antioxidant activities, which were determined by the DPPH_IC50_, ABTS_IC50,_ and TAC tests. The exactitude of this model is validated by correlating experimental data with model predictions, illustrating the effective correlation between them and the viability of the model. In the specific results presented, the formula is a combination of 50% of *M. longifolia*, 27% of *M. aquatica*, 23% *M. suaveolens*. The experimental value for DPPH_IC50_ was seen to be 50.22 ± 0.03 μg/mL, which was quite close to the predicted value of 49.80 ± 0.42 μg/mL, whereas the value of ABTS_IC50_ was established to be 11.90 ± 0.10 μg/mL, which was quite close to the predicted value of 12.08 ± 0.16 μg/mL, whereas the value of TAC was established to be 425.01 ± 4.68 mg AAE/mL EO and predicted value is 429.62 ± 4.14 mg AAE/mL EO.

**TABLE 6:** Predicted and expected responses at the test point for best-fit mixes.

Mixture of combination	Mixture (%)	DPPH_IC50_ (µg/mL)		ABTS_IC50_ (µg/mL)		TAC (mg AAE/mL EO)	
		Exp	Predi	Exp	Predi	Exp	Predi
ML-EO	50%	50.22 ± 0.03	49.80 ± 0.42	11.90 ± 0.10	12.08 ± 0.16	425.01 ± 4.68	429.62 ± 4.14
MA-EO	27%						
MS-EO	23%						

Exp, Experimental; Pred, Predicted.

### 3.8 Computational pharmacokinetic and drug-likeness prediction

**FIGURE 7:**
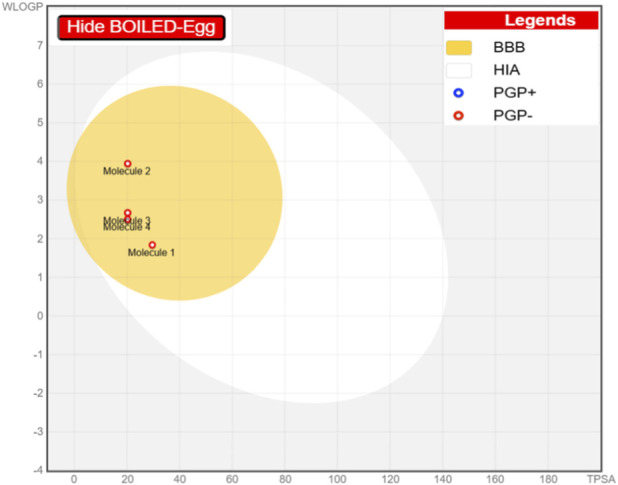
BOILED-Egg model representing the predicted gastrointestinal absorption and blood-brain barrier permeability of Piperitenone oxide (M1), Elemol (M2), Linalool (M3), and α-Terpineol (M4).

The BOILED Egg is a predictive model to understand how the main compounds from the essential oils of the *M. longifolia*, *M. aquatica*, and *M*. *suaveolens* plants work in our body. Figure 7 reveals that the four major molecules, which are piperitenone oxide, elemol, linalool, and α-terpineol, are all in the area of the plot, which means they can easily be absorbed in our stomach and intestines. The four molecules are also in the yolk area of the plot, which is important because it means they can easily get into our brains, which is typical of natural products like monoterpenes and sesquiterpenes that come from essential oils. What is interesting is that the four molecules are in the area of the plot where they’re not likely to be affected by P-glycoprotein.

Even though the molecules come from types of *Mentha* species, they are all close together in the plot. There are some differences, but overall, the main compounds from the essential oils of the *M*. *longifolia*, *M. aquatica*, and *M. suaveolens* plants are good at getting absorbed and getting into our cells.

The physicochemical properties summarized in Table 7 indicate that all four compounds comply with Lipinski’s rule of five, with no observed violations (El Fadili et al., 2023a). Their molecular weights range from 154.25 to 222.37 Da, remaining well below the recommended threshold of 500 Da, which is generally associated with favorable oral bioavailability. In parallel, the calculated LogP values (2.11–3.20) suggest a balanced lipophilicity, sufficient to promote membrane permeability without compromising solubility.

**TABLE 7:** Prediction of physicochemical properties of Piperitenone oxide (M1), Elemol (M2), Linalool (M3), and α-Terpineol (M4).

ML-EO, MA-EO, MS-EO molecules	TPSA	MW	LogP	n-HBA	n-HBD	Violations number
						Lipinski
Rule	<140 Å^2^	<500 D.a	≤5	<10	<5	≤2
M1	29.60	166.22	2.11	2	0	Yes
M2	20.23	222.37	3.20	1	1	Yes
M3	20.23	154.25	2.70	1	1	Yes
M4	20.23	154.25	2.51	1	1	Yes

TPSA, Topological Polar Surface Area; MW, Molecular Weight; n-HBA, Number of Hydrogen Bond Acceptors; n-HBD, Number of Hydrogen Bond Donors.

The topological polar surface area (TPSA) values are relatively low (20.23–29.60 Å^2^), indicating limited polarity and supporting efficient passive diffusion across biological membranes. This is further reinforced by the small number of hydrogen bond acceptors (1–2) and donors (0–1), which remain within the acceptable range for drug-like molecules. Altogether, these parameters highlight a coherent physicochemical profile consistent with small, moderately lipophilic natural compounds typically found in essential oils.

The ADMET predictions presented in Table 8 provide additional insight into the pharmacokinetic behavior of these molecules. All compounds exhibit high predicted intestinal absorption, with values exceeding 93%, suggesting efficient uptake following oral administration. Regarding distribution, the positive LogBB values (0.169–0.625) indicate a capacity to cross the blood-brain barrier, while the LogPS values, although negative, remain within a range compatible with CNS permeability.

**TABLE 8:** ADMET pharmacokinetic features of piperitenone oxide (M1), elemol (M2), linalool (M3), and α-Terpineol (M4).

ADME and toxicity properties														
Models	A	D		M							E	T		
	Intestinal absorption (human)	BBB	CNS	CYP450							Total	AMES toxicity	Hepatotoxicity	Skin sensitization
				2D6	3A4	1A2	2C19	2C9	2D6	3A4				
Unity	(%Absorbed)	(Log BB)	(Log PS)	(Yes NO)							(Log mL min^-1^kg^-1^)	(Yes no)		
Predicted values														
M1	97.685	0.169	−2.769	No	No	No	No	No	No	No	1.152	Yes	No	Yes
M2	93.486	0.625	−2.151	No	No	No	No	No	No	No	1.311	No	No	Yes
M3	93.649	0.608	−2.28	No	No	No	No	No	No	No	0.446	No	No	Yes
M4	94.183	0.305	−2.807	No	No	No	No	No	No	No	1.219	No	No	Yes

From a metabolic perspective, none of the compounds are predicted to act as substrates or inhibitors of major CYP450 isoforms (including CYP2D6, CYP3A4, CYP1A2, and CYP2C9), which may reduce the risk of metabolic interactions and variability. In terms of excretion, the total clearance values show some variability among the compounds, with M3 exhibiting a relatively lower clearance compared to the others, suggesting a potentially longer residence time in the system.

Toxicity predictions reveal that most compounds are non-mutagenic (AMES negative), except M1, which shows a positive prediction and may require further experimental verification. None of the molecules is predicted to be hepatotoxic, which is a favorable indicator for safety (El Fadili et al., 2024). However, all compounds are predicted to cause skin sensitization, a characteristic that is not uncommon for constituents of essential oils and should be considered in their handling and formulation.

Overall, the combined analysis of physicochemical descriptors and ADMET properties suggests that these major constituents share a consistent and favorable pharmacokinetic profile, although certain aspects, particularly related to toxicity, warrant further experimental validation.

**FIGURE 8:**
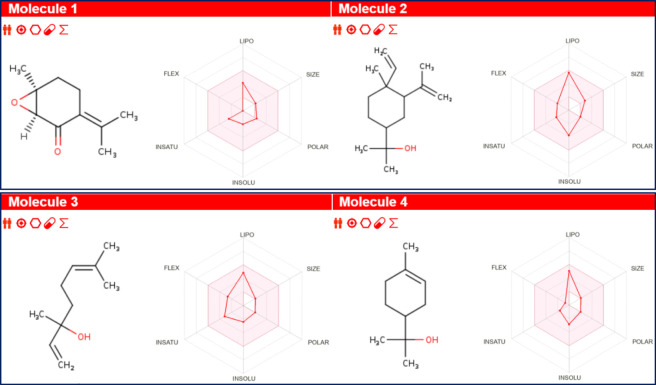
Oral bioavailability radar plots of four major essential oil compounds (M1-M4).

The oral bioavailability of piperitenone oxide, elemol, linalool, and α-Terpineol is very similar, as presented in Figure 8, where the radar plots are all mostly inside the pink area. This means that piperitenone oxide, elemol, linalool, and α-Terpineol all have the physical and chemical properties for the body to absorb them well when taken orally.

This is because piperitenone oxide, elemol, linalool, and α-Terpineol are all made up of terpenes. They are similar in size. Have a moderate ability to dissolve in fats. They are also not very polar, which helps them pass through the body’s membranes easily. Most of these molecules also have a hydroxyl group, which helps balance how well they can dissolve and pass through membranes. Two things that affect how well the body can absorb them orally.

The slight differences are not very big and do not change the overall prediction much. Overall, piperitenone oxide, elemol, linalool, and α-Terpineol all have oral bioavailability profiles. This suggests that they could be useful as bioactive constituents and are worth studying to see if they have any pharmacological effects.

### 3.9 Molecular docking simulations

**FIGURE 9:**
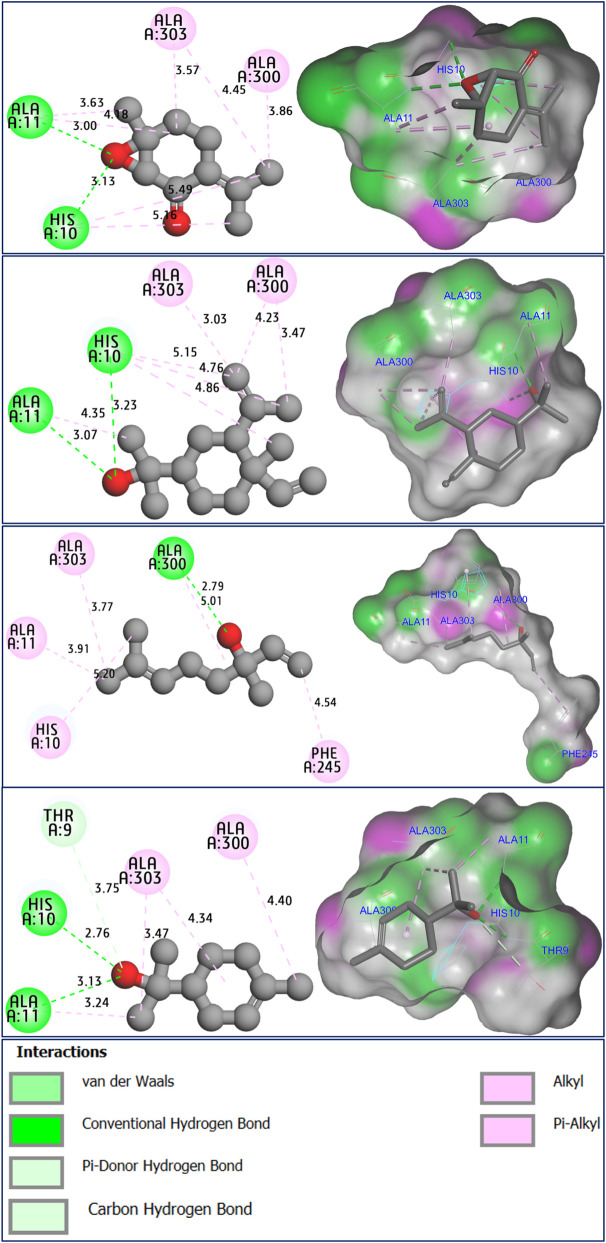
2D and 3D views of intermolecular interactions produced for piperitone oxide, elemol, linalool and α-Terpineol, in complex with NADPH oxidase protein (PDB ID of 2CDU).

Molecular docking results shown in Figure 9 illustrate the inhibition mechanisms of the four main compounds in the investigated oils (piperitenone oxide, elemol, linalool, and α-terpineol) interacting with the targeted NADPH oxidase protein (PDB ID of 2CDU). These compounds fit perfectly into the protein’s binding site through hydrophobic van der Waals interactions, as well as other polar interactions such as hydrogen bonds. This contributes to maintaining their binding to the protein.

Energy analysis of these interactions reveals that all these compounds have a high affinity for the protein. The energy of piperitone oxide is −6.29 kcal/mol, that of elmol is −6.82 kcal/mol, that of linalool is −5.29 kcal/mol, and that of α-terpineol is −6.24 kcal/mol. This means that all these compounds tend to bind to the protein, but Elmol is the most readily available. This compound has high energy, indicating a strong interaction with the protein.

Piperitone oxide acts by binding to a hydrophobic site in the protein. It relies on van der Waals interactions with certain protein fragments such as ALA11, ALA300, and ALA303, as well as a hydrogen bond with another protein fragment. This helps stabilize piperitone oxide within the protein. Elmol has a higher protein-binding affinity. It binds completely to the protein’s active site and relies on numerous hydrophobic interactions with fragments like ALA300 and ALA303. It also utilizes hydrogen bonds, further enhancing its binding activity. This is why elemol is more energetic and binds strongly to proteins.

Linalool, on the other hand, is slightly different. It interacts with proteins in a different way. Its binding relies primarily on van der Waals interactions with hydrophobic protein fragments such as ALA300 and ALA303, in addition to a hydrogen bond. Linalool’s protein binding is less robust than that of other compounds. Linalool’s energy is slightly higher than that of other compounds, meaning it does not bind to proteins as readily. α-Terpineol, on the other hand, interacts with the protein in a specific manner. It utilizes hydrophobic interactions with components such as ALA11, ALA300, and ALA303, and also forms a hydrogen bond with a polar region of the protein. This enhances its binding affinity. α-Terpineol’s energy is close to that of piperitinone oxide, indicating that it binds to the protein in a specific way.

In summary, these four compounds, piperitenone oxide, elemol, linalool, and alpha-terpineol, all interact specifically with the NADPH oxidase protein. This interaction results from a combination of hydrophobic and polar interactions. Elemol is the most effective protein-binding agent, closely followed by piperitinone oxide, and then α-Terpineol. Linalool, while less effective, is still a significant activator.

## 4 Conclusion

This is the first time the combination of three essential oils from *M. longifolia*, *M. aquatica,* and *M. suaveolens* was subjected to optimization via simplex-lattice design to enhance antioxidant activity. The optimal mixture was determined to contain 50% ML-EO, 27% MA-EO, and 23% MS-EO. The IC_50_ values were 50.22 μg/mL for DPPH, 11.90 μg/mL for ABTS, and 425.01 mg AAE/mL EO for TAC, with a desirability rating of 0.99. These optimal formulations were validated experimentally, exhibiting less than 5% deviation from the calculated IC_50_ values (49.80 μg/mL DPPH; 12.08 μg/mL ABTS; 429.62 mg AAE/mL EO TAC). The results of *in silico* ADMET properties, demonstrated that the studied essential oils exhibit favorable pharmacokinetic profiles and good oral bioavailability. This novel approach has provided a step towards addressing a previously unreported area in literature and developing naturally occurring antioxidants agents. Future research should include molecular dynamics studies to better understand whether these compounds bind to affect the target enzymes of interest, along with evaluating all aspects required to confirm the therapeutic efficacy of the optimized formulation.

## Data Availability

The original contributions presented in the study are included in the article; further inquiries can be directed to the corresponding author.
